# Developing Theoretical Models for Atherosclerotic Lesions: A Methodological Approach Using Interdisciplinary Insights

**DOI:** 10.3390/life14080979

**Published:** 2024-08-05

**Authors:** Amun G. Hofmann

**Affiliations:** FIFOS—Forum for Integrative Research & Systems Biology, 1170 Vienna, Austria; amungeorg.hofmann@gesundheitsverbund.at

**Keywords:** atherosclerosis, logistic map, ecology, disease modeling, Markov model

## Abstract

Atherosclerosis, a leading cause of cardiovascular disease, necessitates advanced and innovative modeling techniques to better understand and predict plaque dynamics. The present work presents two distinct hypothetical models inspired by different research fields: the logistic map from chaos theory and Markov models from stochastic processes. The logistic map effectively models the nonlinear progression and sudden changes in plaque stability, reflecting the chaotic nature of atherosclerotic events. In contrast, Markov models, including traditional Markov chains, spatial Markov models, and Markov random fields, provide a probabilistic framework to assess plaque stability and transitions. Spatial Markov models, visualized through heatmaps, highlight the spatial distribution of transition probabilities, emphasizing local interactions and dependencies. Markov random fields incorporate complex spatial interactions, inspired by advances in physics and computational biology, but present challenges in parameter estimation and computational complexity. While these hypothetical models offer promising insights, they require rigorous validation with real-world data to confirm their accuracy and applicability. This study underscores the importance of interdisciplinary approaches in developing theoretical models for atherosclerotic plaques.

## 1. Introduction

Atherosclerosis can be characterized as a nonlinear dynamical system. It is nonlinear because its plaque development, including both build-up and rupture, is ill-described by laws of proportionality, a fact that has been previously used to model the disease [1,2] and is not surprising considering that most systems found in nature are inherently nonlinear [3]. Atherosclerotic lesions are initiated by the subendothelial retention of lipoproteins such as low-density lipoprotein (LDL), which obtain proinflammatory and immunogenic features by oxidation. This subsequently induces inflammatory cell recruitment and the formation of foam cells [4]. The following multifactorial cascade is a complex interplay of several pathophysiological processes and includes apoptosis, vascular smooth muscle cell proliferation, calcification, erosion or rupture, and thrombosis [5]. Clinically, the disease is frequently classified as asymptomatic or symptomatic regardless of its location, which, in the case of coronary arteries, can range from angina pectoris to myocardial infarction [6], or can refer to stroke stemming from atherosclerotic carotid artery stenosis [7]. The difference between clinically silent disease and symptomatic events implies significant differences in both treatment approaches and patient outcomes. Our extent of understanding atherosclerosis is innately linked to our therapeutic abilities. Mathematical modeling approaches to the disease can aid the former to expand the latter. Historically, several attempts have been made with increasing complexity and accuracy [8,9]. The guiding proposition for efficient and accurate modeling is the prediction of plaque development, thereby enabling the stratification of patients at an elevated risk for symptomatic events [10].

Mathematical, computational, and statistical models are ubiquitous in quantitative research and are frequently used to either study associations or facilitate predictions. Models convert real-life situations and problems into mathematical form, giving researchers the opportunity to transfer solutions from mathematical equations to the practical problem at hand [11]. The importance of mathematical models in biomedicine has been discussed previously, but so have the frequently observed difficulties in establishing an interdisciplinary foundation between modelers and biomedical researchers to efficiently develop such models [12]. A potential approach could be the transfer of already established models from distinct research areas based on the presence of analogies.

Compartmental models, such as those based on ordinary differential equations, have been employed to simulate the temporal progression of atherosclerosis by considering the interplay of different biological variables. While these models offer a temporal perspective, their ability to capture the spatial distribution of plaque characteristics remains limited. Moreover, atherosclerosis is inherently heterogeneous, with variations in the plaque composition and stability occurring at distinct locations within the vasculature.

Atherosclerotic lesions are never under continuous (24/7) surveillance. Depending on their location and the disease progression, surveillance using imaging is conducted at fixed time intervals (discrete time points). Continuous modeling approaches might, therefore, enhance our ability to investigate the pathology, but they are difficult to combine with and validate by clinical data. Furthermore, the localized nature of plaque progression, coupled with the influence of neighboring regions on stability or rupture potential, necessitates an evolution in modeling approaches. In the realm of mathematical modeling, the Markov chain has emerged as a powerful tool for capturing dynamic processes in various research fields. Its applications range from epidemiology and finance to ecology, demonstrating versatility in modeling transitions between different states over time. In the context of atherosclerosis, where disease progression involves transitions between stable and unstable plaque states, the Markov chain offers a promising approach to simulate and predict the likelihood of symptomatic events.

The following manuscript works through two examples to illustrate a framework for the process of model transfer in atherosclerosis research. The first model (logistic map) is simplistic but inherently captures properties that correspond to atherosclerotic plaques, and such models might assist in expanding our understanding of pathologies and their courses. The second model (Markov model) is an example of how analogies can be used to establish a simple and accessible model and, subsequently, build it up step-by-step to capture and reflect more complex aspects of a disease.

## 2. Logistic Map

### 2.1. Logistic Map

**Figure 1:**
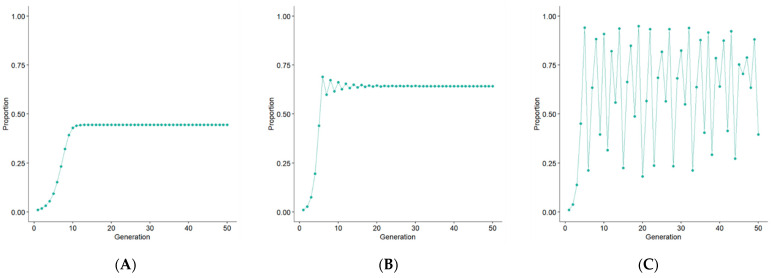
Examples of the behavior of the logistic map for different values of *r* – 1.8 (**A**), 2.8 (**B**), and 3.8 (**C**). (*t*_0_ = 0.1).

The logistic map is a polynomial mapping frequently used to illustrate how simple nonlinear models can result in complex behavior and chaos [11]. It is a first-order difference equation used for discrete time population modeling analogous to the continuous logistic differential equation and characterized by the equation:

(1) $$P_{t+1}=r*P_{t}(1-P_{t})$$

where *P* is the proportion of the potential maximum population size, either at time *t* or *t* + 1, and *r* is a growth parameter that determines the population change. Therefore, *P* must be a number between 0 and 1 and can be regarded as a proportion of the maximum achievable population size. The behavior of the function is essentially dependent on the value of *r*. For any value between 0 and 1, the population will eventually become 0, while between 1 and 2, it will converge to $\frac{r-1}{r}$, regardless of the initial population size. With *r* between 2 and 3, the function starts to exhibit more complex behavior, and while it still converges to $\frac{r-1}{r}$, it first fluctuates around the value for several generations. For any value above 3, oscillations will arise, initially between two values, then four, and at *r* ≈ 3.56995, the function starts to exhibit chaotic behavior [13] (see Figure 1A–C).

The logistic map has further properties such as attractors that are especially well-illustrated by bifurcation diagrams, but are beyond the scope of this work.

### 2.2. Translation to Atherosclerotic Plaques

Instead of treating *P* from Equation (1) as a population size, one can regard *P* as the proportion of the area of an arterial lumen *A* that is occupied by an atherosclerotic plaque. However, what standard applications of the logistic map in ecology commonly do not take into account is an increase in the maximal achievable population size. Atherosclerotic lesions do have this property, as positive remodeling inflicted in the arterial wall in the initial disease stages leads to an increase of the luminal area to maintain the perfused lumen. Therefore, for a distinct number of timepoints *i*, *A* expands from *A*_0_ at *t*_0_ (the initial arterial lumen) until it converges to a maximum area *A_max_*,

(2) $$\underset{t_{0}{\to t}_{i}}{\lim}A_{0}=A_{max}$$

### 2.3. Growth Parameter r as an Aggregate Function

The growth of an atherosclerotic plaque is dictated by a plethora of parameters, including the availability of lipoprotein particles, macrophage influx rate, cytokine production [2], or the clearance rate of dead cells and debris [14], for example. In the present model, *r* is proposed as an aggregate function of all factors contributing to plaque development and clearance, i.e., *r* is the net growth rate determined by the inter- and counterplay of all biological processes involved in atherosclerotic lesion development. In ecology, *r* is also an aggregate for an array of modifiers such as food supply, predator–prey relationships, and climate. However, as opposed to conventional population modeling, *r* is not a fixed value, but itself a time-dependent function with its own dynamic, leading to:

(3) $$\frac{dr}{dt}\neq 0$$

The function *r*(*P*) is shown as Equation (4) and has several distinct properties:

(4) $$r\left(P\right)=\alpha *(1+\beta_{1}*f1\left(P\right)+\beta_{2}*f2\left(P\right)+\beta_{3}*f3\left(P\right)+\beta_{4}*f4\left(P\right))$$

where α is the baseline growth rate, *f*(*i*)(*P*) are functions representing specific factors affecting atherosclerotic disease, and *β_i_* is the weighting coefficient that determines the relative importance of each factor. These factors could, for example, reflect hemodynamic, inflammatory, mechanical, and metabolic influences that are determined by a magnitude of variables, with examples listed in Table 1.

### 2.4. Implementation of the Logistic Map in Atherosclerosis

**Figure 2:**
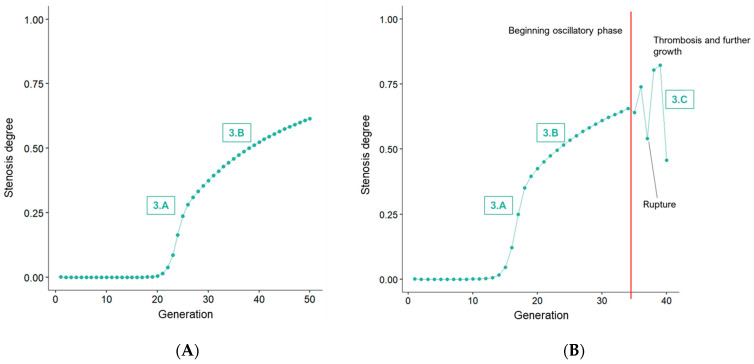
Hypothetic models of a stable, asymptomatic plaque (**A**) and an unstable, potentially symptomatic plaque (**B**). A–C refer to corresponding plaque illustrations in Figure 3. Parameter *r* increases in both examples, by a constant factor for each generation (0.05 in (**A**) vs. 0.08 in (**B**)). The red line in (**B**) marks the beginning of the oscillatory phase that reflects a disease stage where patients are at risk of cardiovascular events due to unstable plaques.

The dynamic property of *r*(*P*) reflects the natural course of the disease, which can progress quicker or slower, depending on the modification of the involved processes. For example, statins reduce LDL-C serum levels, thereby creating a negative stimulus for *r*. Nevertheless, once an atherosclerotic plaque forms at a location of damaged endothelium, a predilection site for the retention of lipoproteins and, therefore, further progression has been established, i.e., plaque attracts the build-up of more plaque. As seen clinically, lesions commonly, at best, stabilize, even though regression is theoretically feasible [15]. Therefore, in atherosclerosis, *r* frequently has a lower bound of 1 and stable plaques might be characterized by convergence to a boundary upper threshold (see Figure 2A). However, more clinically relevant are unstable plaques that can lead to symptomatic disease manifestations.

### 2.5. Identifying Patients at Risk for Symptomatic Disease

**Figure 3:**
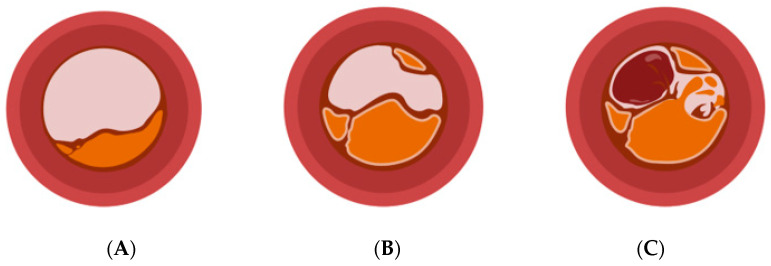
Different disease stages of an atherosclerotic plaque. (**A**) Depicts an early lesion with minimal luminal reduction, (**B**) a further developed but asymptomatic manifestation, and (**C**) an unstable plaque that is characterized by progressed luminal reduction, rupture, and subsequent thrombosis (Created with BioRender.com).

Unstable plaques are defined by values of *r* > 2. A reduction in *P* indicates the beginning of oscillations observed at this growth rate. This, in turn, corresponds to plaque erosion or rupture, increasing the lumen size—if only for a thin layer of cells, as seen for cap erosion—but, at the same time, exposing the thrombogenic material inside the core and resulting in thrombosis, which, in turn, can cause both thrombotic occlusion or embolization to more peripheral arteries. Correspondingly, healing plaques have been observed to incorporate thrombi, thereby significantly increasing in size, also referred to as constrictive remodeling [7], which is ultimately captured by a subsequent increase in *P* that equals the oscillatory phase at *r* > 2 (see Figure 2B). The oscillatory phase in the model correlates with observations from clinical studies that have reported repeated erosion/rupture and healing with accelerated luminal stenosis prior to clinically detectable events [16,17,18]. Illustrations of atherosclerotic plaques in different development stages are shown in Figure 3. Within the proposed model, the distinction between symptomatic and asymptomatic disease can, therefore, be illustrated by margins of *r*, i.e., net plaque progression, which leads to the question of how this can translate into clinics and augment risk stratification protocols.

### 2.6. Stenotic Degree and Symptomatic Disease in the Logistic Map

**Figure 4:**
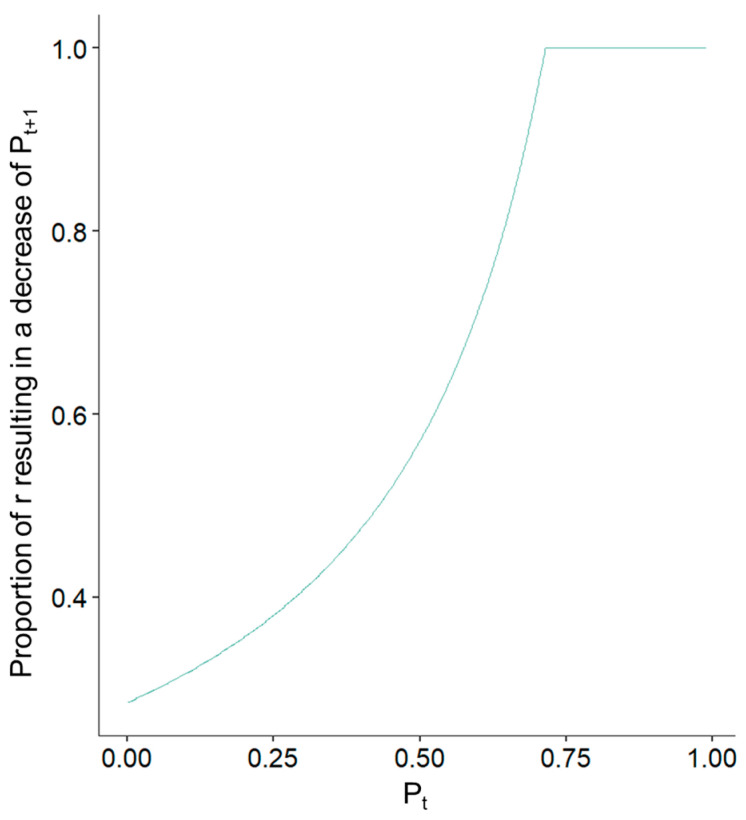
Figure 4 illustrates, for a given value of *P_t_* in the logistic map, the proportion of values of *r* resulting in a subsequent decrease in *P*_*t*+1_. As *P* increases, the proportion of potential values of *r* (under the boundaries of 0 and 3.5, where the function always exhibits chaotic behavior) leading to a decrease in *P* in the subsequent generation increases approximately exponentially. This corresponds to the observation that bigger atherosclerotic plaques are more likely to cause cardiovascular events.

The model also captures well why plaques of a higher stenotic degree are more likely to induce clinical cardiovascular events. Atherosclerotic plaques become more sensitive and vulnerable to rupture as they increase in size. With an increase in *P*, the proportion of potential values of *r* resulting in *P_t_*_+__1_ < *P_t_* increases approximately exponentially until reaching its maximum of 1 around 0.7. As *P* increases, there are less and less values for *r* that fulfill the condition *P_t_* < *P_t_*_+1_. In other words, the logistic map inherently becomes unstable in that region, and the bigger *P_t_* becomes, so does the probability of instability depicted by *P_t_*_+1_ < *P_t_* (see Figure 4). This would indicate that, around a stenosis of 70%, ruptures or at least erosions become inevitable and correspond to the clinical guideline recommendation for prophylactic atherectomy in the case of 70% asymptomatic carotid artery stenosis due to its elevated risk of causing events. Similarly, 70% stenosis is frequently considered as severe in coronary arteries and prone to induce myocardial infarction [19]. However, this does not mean that every rupture or erosion predicted under the logistic-map-based model must result in a clinically observable event, i.e., not every plaque with a stenotic degree >70% must become symptomatic. It has been previously observed that, while most clinical events stem from ruptures, not all ruptures must cause an event (the difference between necessary and sufficient conditions) [20].

### 2.7. Introducing Imaging Data

An atherosclerosis model based on the logistic map not only facilitates the simplification of a complex pathophysiological process, but also has the potential for accelerated translation to clinics. Patients at risk of symptomatic disease could be identified by approximating the dynamics of atherosclerotic plaques in distinct sites based on planimetric measurements of medical imaging technologies. For carotid artery stenosis, this could be achieved by repeated ultrasound investigations, which is already the standard surveillance technique [9]. Consecutive ultrasound studies could be entered into Equation (1) for *P* and solved for *r*. If *r* should be found growing towards >2, these patients could enter different, i.e., more frequent, surveillance protocols, or might receive prophylactic endarterectomy based on their plaque growth dynamics instead of hemodynamically inferred stenosis degree, as is currently the state-of-the-art practice [7]. While efficient and accurate non-invasive and radiation-independent imaging of the coronary arteries is still subject to more challenges compared to carotids, further technological advances are to be expected that could offer similar potential regarding risk stratification.

## 3. Markov Models

While the logistic-map-based model draws its inspiration directly from population modeling in ecology, Markov models have already been employed and investigated in various fields. In medicine, they have been especially popular in decision-making processes [21,22], but have also been investigated to predict disease progressions [23]. Outside of biomedical research, Markov models are prominent tools for avalanche or rockslide predictions, especially in the form of hidden Markov models [24]. Analogous to atherosclerotic plaques, they are characterized by switching from stable to unstable states with the local spatial dependencies of neighboring points. A potential path to develop a Markov model for atherosclerotic plaques based on this analogy is illustrated below, progressing from a simple Markov chain to more complex spatial models.

### 3.1. Markov Chain Model

Model development is initiated with a simple Markov chain. A traditional Markov chain model models the transitions between different states of a system, in this case, plaque stability (stable or unstable). The model incorporates variables such as inflammation levels (*I*), lipid content (*L*), shear stress (*S*), and plaque burden (*B*).

*P*(*_Stable_*_→*Unstable*_) = *Base Probability* + *w*1⋅*I* + *w*2⋅*L* + *w*3⋅*S* + *w*4⋅*B* (5)

where *w*1, *w*2, *w*3, and *w*4 are the weights for inflammation, lipid content, shear stress, and plaque burden, respectively, with a similar purpose as the beta coefficients in (4). Adjusting these weights based on empirical data is crucial to establish an accurate model that considers the impacts of various pathophysiological factors on the probability of transitioning from a stable to unstable plaque.

### 3.2. Spatial Markov Model

Recognizing the spatial heterogeneity of atherosclerotic plaques, the Markov chain framework is extended to a spatial Markov model. In this model, the probability of transitioning between states is not only influenced by the current state, but also by the states of neighboring plaque regions. These spatial dependencies are represented using a spatial dependence matrix, which quantifies the influence of each neighboring point on the focal point.

*P*(*_Stable_*_→*Unstable*_) = *Base Probability* + *w*1⋅*I* + *w*2⋅*L* + *w*3⋅*S* + *w*4⋅*B* + *w*5⋅*D* (6)

where *w*1, *w*2, *w*3, *w*4, and *w*5 are the weights for inflammation, lipid content, shear stress, total plaque burden, and spatial dependence, respectively. *D* represents the contribution from the spatial dependence matrix.

### 3.3. Markov Random Field

Additionally, the application of a Markov random field is illustrated to capture more complex spatial interactions. The Markov random field incorporates an energy function that considers the joint probabilities of neighboring points, allowing for a more nuanced representation of spatial dependencies.

### 3.4. Transition Probabilities in Markov Chain Models

The Markov chain model reveals the transition probabilities between stable (*S*) and unstable (*U*) plaque states. Denoting the transition probability from state *i* to state *j* as *P_ij_*, the model yields:

*P_S_*_→*U*_ = *α_SU_* (7)

*P_U_*_→*S*_ = *α_US_* (8)

where *α_SU_* and *α_US_* are the transition rates from stable to unstable and unstable to stable, respectively.

### 3.5. Stability Assessments

The model incorporates stability assessments based on the stationary distribution, calculated as the eigenvector corresponding to the eigenvalue 1 of the transition probability matrix. The stationary distribution (π) yields:

π = [π_S_, π_U_] (9)

The stationary distribution represents the long-term distribution of the system’s states. In other words, as time extends to infinity, the probabilities of being in each state stabilize, and this stabilized distribution is the stationary distribution. For a Markov chain, if the system is irreducible (a positive probability of moving from any state to any other state) and aperiodic (no regular pattern in the transitions), it has a unique stationary distribution. This stationary distribution is associated with an eigenvalue of 1. The eigenvector corresponding to this eigenvalue equals the long-term probabilities of being in each state. π, in the context of Markov chains, represents the stationary distribution vector. Each element of this vector corresponds to the long-term probability of being in the respective state. Assuming a two-state system (e.g., stable and unstable plaque states), π = [π_S_, π_U_], where π_S_ is the probability of being in the stable state in the long run and π_U_ is the probability of being in the unstable state.

### 3.6. Prediction of Atherosclerotic Events

Using the transition probabilities enables predictions of the occurrence of atherosclerotic events over time. The probabilities of remaining in the stable state (*P_S__*→*S_*) and transitioning to the unstable state (*P_S__*→*U_*) at time *t* are expressed as:

*P_S_*_→*S*_ = *e*^(−^*^𝛼SUt^*^)^ (10)

*P_S_*_→*U*_ = 1 − *e*^(−^*^𝛼SUt^*^)^ (11)

### 3.7. Spatial Transition Probabilities in a Spatial Markov Model

Incorporating spatial dependencies, the spatial Markov model introduces a spatial dependence matrix (*D*), influencing the transition probabilities. The spatial transition probability matrix (*P_Spatial_*) is defined as:

*P_Spatial_* = *D* ⊙ *P_Markov_* (12)

where ⊙ represents the element-wise Hadamard product, combining the spatial dependence matrix with the Markov chain transition probability matrix. In the context of the spatial Markov model, the spatial dependence matrix (*D*) represents the matrix capturing the spatial relationships or dependencies between different points on the plaque surface. Each element *d_ij_* of this matrix indicates the strength or nature of the spatial dependence between points *i* and *j*. This matrix represents the transition probabilities between different states in the Markov chain model—*P_Markov_*. Each element *p_ij_* of this matrix represents the probability of transitioning from state *i* to state *j*.

Combining the spatial dependence matrix (*D*) with the Markov chain transition probability matrix (*P_Markov_*) using the Hadamard product involves multiplying the corresponding elements of these matrices. The resulting matrix, *P_Spatial_*, is a modified transition probability matrix that accounts for spatial dependencies.

The spatial dependence matrix influences the transition probabilities between different states based on the spatial relationships between points on the plaques surface. This modification is important for capturing how the stability of one region may be influenced by the stability of its neighboring regions.

### 3.8. Spatial Heatmap Visualization

**Figure 5:**
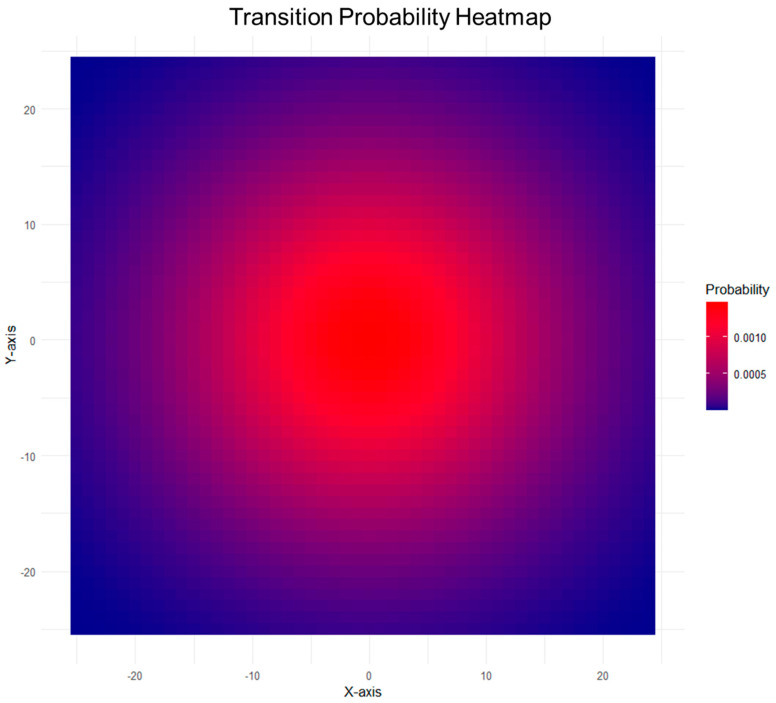
Figure 5 illustrates how spatial Markov models can be used to assemble heatmaps visualizing the probability of a plaque area to become unstable and thereby more likely to cause symptomatic events. A three-dimensional plaque can be visualized in a two-dimensional heatmap analogous to the flattened plaque surface (red indicating higher probability, blue lower probability).

The spatial Markov model’s outcomes can be visually represented through heatmaps, illustrating the spatial distribution of the transition probabilities across the plaque surface. Points with higher probabilities are denoted by warmer colors, emphasizing regions of an increased susceptibility to instability (Figure 5).

### 3.9. Markov Random Field Model and Energy Function

The Markov random field introduces an energy Function (*E*) that captures the joint probabilities between neighboring points. The energy function is defined as:

*E*(*config*) = ∑*_i_V*(*state_i_*) + ∑*_i,j_U*(*state_i_*, *state_j_*) (13)

where *V* is the potential function for each individual point and *U* is the potential function representing the interactions between neighboring points.

### 3.10. Transition Probability Matrix:

The transition probability matrix (*P_MRF_*) is derived from the energy function and used to calculate the probability of transitioning between states. The matrix is expressed as:

*P_MRF_* ∝ *e*^−*E*(*config*)^ (14)

## 4. Discussion

This work illustrates how heuristics and interdisciplinary approaches to disease modeling can be transferred to atherosclerosis research to enhance our ability to investigate pathologies. The first model (logistic map) was primarily developed to model population dynamics in ecology, but as illustrated, incorporates several properties that correspond to atherosclerotic plaques, mainly their chaotic behavior and instability. The second string of models (Markov models) are examples of how a simple model that is derived from a mechanistic analogy can be further built up to capture the more complex nuances of a pathology. Both might be reasonable strategies to expand the toolkit of available models in atherosclerosis research.

Previous efforts to model symptomatic atherosclerotic manifestations have primarily focused on individual risk factors, clinical variables, and statistical methods to correlate these risk factors with the likelihood of adverse cardiovascular events. While these models have provided valuable insights and improved patient care, they lack the accessibility to be directly transferred to clinical practice and frequently the spatial resolution needed to discern localized variations in plaque vulnerability.

There are several advantages of a modeling approach for atherosclerotic lesions that is built around the logistic map. The discrete time model fits the surveillance protocols used in clinical practice well, where patients are examined based on fixed intervals, e.g., every 6 or 12 months. Under the proposed model, each visit would constitute a data point analogous to generations in a population. The logistic map inherently captures nonlinear dynamics, which is necessary for complex biological processes like atherosclerosis. The ability of the logistic map to exhibit chaotic behavior under certain conditions might be a desirable characteristic when modeling something as complex as plaque progression. Its underlying function is also simple if not intuitive for non-mathematicians, which promotes interdisciplinary exchange. Finally, expressing the growth rate as an aggregate function of relevant risk factors allows for flexibility and adaptability to the multifactorial nature of atherosclerotic plaques based on available data.

A limitation of the model as proposed is the dimensionality reduction of a three-dimensional atherosclerotic lesion to a two-dimensional section. However, a transition to a more complex model could be achieved by regarding the plaque as a succession of finite sections. Clinically, dynamics could be investigated for two-dimensional sections of a thickness that is determined by the resolution of the used imaging modalities, in the case of ultrasound, for example, around 2 mm when using a perpendicular angle [25]. With the increased implementation of three-dimensional imaging studies such as 3D ultrasound, the proposed planimetric approach might be superseded by a volumetric approach, in which a complete plaque at a defined anatomic location is investigated.

Furthermore, atherosclerotic plaques exhibit spatial heterogeneity, and the local interactions between neighboring regions influence the stability and rupture potential of the plaque cap. Neglecting such spatial dependencies may limit the accuracy and clinical relevance of predictive models. Comparing the Markov chain and spatial models, several advantages of the spatial approach exist. Spatial models capture local interactions and dependencies, providing a more nuanced depiction of plaque dynamics. The traditional Markov chain, while easily interpretable, might overlook and oversimplify critical spatial considerations, limiting its applicability in a complex biological system. Distinguishing between the spatial Markov and Markov random field models, the former demonstrates simplicity and ease of interpretation, making it more accessible for clinical application. The Markov random field, while offering detailed insights into spatial interactions, presents challenges in parameter estimation and computational complexity. Balancing model complexity with practicality is crucial for promoting translational research. Spatial models have the ability to model local interactions within the plaque microenvironment. The inclusion of spatial dependence matrices and interaction potentials facilitates the identification of regions with a heightened susceptibility to instability. This capability is essential for a further transition to precision medicine, where interventions at distinct high-risk sites may be more effective than heuristic approaches that are based on the degree of stenosis. The spatial Markov model, visualized through heatmaps, provides a spatially explicit representation of transition probabilities. Identifying spatial patterns enhances our ability to discern the areas of the plaque with elevated risk, offering a valuable tool for risk stratification and personalized treatment strategies.

The validation of both a logistic-map-based mode as well as spatial models demands comprehensive datasets, including longitudinal imaging studies at regular intervals, ideally starting from small lesion sizes over the course of years and preferably decades, and detailed spatial information, including high-resolution imaging and multi-dimensional clinical variables. Meeting these data requirements poses challenges, particularly in large-scale clinical studies. The computational demands of spatial models, especially the Markov random field, may hinder real-time applications and limit their feasibility in clinical practice. Addressing computational challenges is crucial for translating these models into practical tools for healthcare providers.

In general, mathematical models, so far, have assisted greatly in increasing our understanding of the development and progression of atherosclerotic lesions, including the involved inflammatory process [26] and hemodynamic changes associated with the disease [27]. More specifically, the nonlinear dynamics and staple paradigms of their study, including bifurcations [28] and chaos [29], have been previously used to model their pathology. However, historic approaches might be difficult to translate into clinical practice, considering the necessity to implement parameters that are often difficult to quantify, such as chemokine production or the clearance rates at the core of the lesion. Additionally, spatial models offer another promising avenue for personalized risk assessment in atherosclerotic cardiovascular disease. By incorporating spatial dependencies, these models enhance the accuracy of risk predictions, enabling clinicians to tailor interventions based on individual plaque characteristics. Understanding the spatial patterns of instability allows for targeted interventions. Clinicians can focus on regions with higher predicted probabilities of instability, potentially preventing atherosclerotic events through localized therapeutic approaches.

While both presented models, therefore, have certain advantages and limitations, especially for timely validation using patient data, the focal point of this work is the so far scarcely explored approach for developing and improving pathology models by incorporating interdisciplinary methods. Pragmatic approaches might both offer new insights, as well as better translation into clinical practice, than very complex models.

## Figures and Tables

**Table 1:** Functions and their variables that could be used to assemble an aggregate growth function *r(P)*.

	Function	Variables
Hemodynamic factors	f1(*P*)	Blood pressure Flow pattern Shear stress Wall pressure
Inflammation	f2(*P*)	Macrophage influx Clearance rate Cytokine production Lymphocyte activation
Mechanical factors	f3(*P*)	Plaque composition Plaque geometry Fibrous cap thickness
Metabolic factors	f4(*P*)	Lipoprotein particle availability Lipoprotein influx Oxidative stress

## Data Availability

The data presented in this study are available on request from the corresponding author.
